# Laser Scanning and Parametrization of Weld Grooves with Reflective Surfaces

**DOI:** 10.3390/s21144791

**Published:** 2021-07-13

**Authors:** Andrej Cibicik, Lars Tingelstad, Olav Egeland

**Affiliations:** Department of Mechanical and Industrial Engineering, Norwegian University of Science and Technology (NTNU), 7034 Trondheim, Norway; lars.tingelstad@ntnu.no (L.T.); olav.egeland@ntnu.no (O.E.)

**Keywords:** structured light sensor, weld groove, parametrization, feature extraction

## Abstract

This paper presents a novel weld groove parametrization algorithm, which is developed specifically for weld grooves in typical stub and butt joints between large tubular elements. The procedure is based on random sample consensus (RANSAC) with additionally proposed correction steps, including a corner correction step for grooves with narrow root weld, and an iterative error elimination step for improving the initially obtained data fit. The problem of curved groove sides (due to the pipe geometry) is attributed and solved. In addition, the procedure detects and eliminates several types of data noise due to laser line reflections. The performance of the procedure is studied experimentally using small-scale test objects, which have been ground using typical industrial power tools to achieve a realistic level of reflections. The execution times and data fit errors of the proposed procedure are compared to a procedure based on a more conventional RANSAC approach for line segment detection.

## 1. Introduction

Welding is an important manufacturing process for steel components and structures. Welding is often done by manual labor, especially for complex welding processes, complex interface geometries, and welds with high quality requirements. Alternatively, welding automation using robotic arms (i.e., robotic welding) can be used for products welded at factories. Robotic welding is relatively easy to implement for production lines with standard products with known geometry and small welds with few beads in the weld cross-section. However, robotic welding becomes more challenging when large components have to be welded together. This is due to larger geometrical deviations of components and large welds required to connect them. In such applications, scanning and parametrization of weld grooves is a vital step before welding can start. In this work, we propose an algorithm for precise parametrization of weld grooves at interfaces between large tubular joints when a 2-dimensional (2D) laser scanner is used.

Various types of sensors can be used in robotic welding. The common application areas are seam tracking, weld pool monitoring, quality inspection, weld position identification, and groove geometry extraction. When the set of parameters in the extracted features is sufficient to describe a weld groove section uniquely, then the process of feature extraction can also be referred to as weld groove parametrization. Typical sensor technologies are ultrasound sensors [1], infrared sensors [2], arc-sensing [3], magneto-optical sensors [4], and computer vision sensors [5,6].

Computer vision sensors are often used for weld groove (or weld seam) tracking, feature extraction, or groove geometry parametrization. Although, in some applications, analysis of global 3-dimensional (3D) point clouds [7,8,9] or passive light systems [10] can be used for global weld seam detection, most of the research in weld seam tracking and groove feature extraction is done using local structured light sensors. That is, sensors projecting structured light into the region of interest (ROI). Structured light sensors are especially efficient when high-precision groove parametrization is required [11]. The reader can find more information on the application of different structured light sensors to robotic welding in the recently published review article [5].

Line laser triangulation sensors are a type of structured light sensors where the projected light is a line. Such sensors can scan one weld groove section at a time. The groove parametrization task can then be defined as a replacement of all scanned data points with a small set of parameters. One reasonable set of such parameters are the coordinates of corner points in the groove section [12]. A simple way to detect the corners is to identify points where the direction of the profile changes by using the gradient. This was done in [13], where the authors applied the technique to V-grooves (or U-grooves). A similar feature extraction technique for V-grooves using averaged slope between four consequent points was presented in [14], where the least-square method was additionally used to improve the location of corner points. Another corner detection algorithm based on the first and second derivatives with the Gaussian filter was suggested in [15]. Alternatively, a template matching method can be used for groove geometry parametrization. The method allows for missing data recovery, which is especially important for missing corners of a V-groove [16]. Parametrization of weld grooves can also be done using random sample consensus (RANSAC) [10,17,18]. The groove corner detection based on RANSAC consists of the following steps. First, a point pair is randomly selected from the entire dataset, then the points close to the line (inliers) through the point pair are identified. These two steps are repeated for several iterations until the fit with the most inliers is found, then a line segment is defined using the best inlier set. Once all line segments are found, then groove corners are found as intersections between the line segments.

When weld grooves in large tubular joints are scanned and parametrized, two main joint types should be taken into consideration. These are butt joints with a typical V-groove and stub joints (or T-joints) with an asymmetric weld groove with one side being curved (due to the pipe geometry) [19]. The online weld seam tracking technique was proposed and tested for large tubular joints in [20], where the pipes were interfaced and welded with a fillet weld (i.e., without a pre-machined weld groove). A similar work for the intersection between a pipe and a sphere was presented in [21], where the problem of curved groove sides was discussed. In the case of multi-pass welding, the groove around the whole joint should be first scanned and parametrized offline before the welding can start. This is due to non-uniform welding grooves with a varying number of beads required in each scanned groove section [22,23,24].

Commonly, weld grooves are ground before welding, which means that the groove steel surface can be very reflective, and the groove parametrization algorithms have to be able to handle some data noise. When laser triangulation sensors are used, noise can be handled at two levels: the camera image level and the point cloud level [25]. If there is access to raw image data, noise filtering at the image level is possible [14,16,26,27]; alternatively, noise can be removed at the level of the final dataset (the triangulated 2D point cloud level) [13,28]. The latter case is more practical when the commercial sensors with partially closed interfaces are used.

Although a lot of research has been done on weld groove (or weld seam) parametrization (i.e., feature extraction), most of the research has dealt with V or U-grooves. In addition, little attention had been paid to the cases with strong surface reflections and high levels of data noise. In this paper, we propose a novel procedure for parametrization of weld grooves commonly used in stub or butt joints of large tubular elements. The algorithm takes a triangulated 2D point cloud from a commercial line laser sensor as an input and returns the corners points of a groove. The proposed procedure is based on a RANSAC search, where we propose several additional correction steps to obtain a better data fit and enable effective performance for grooves with narrow root welds. The procedure can also detect some forms of data noise caused by laser line reflections on the ground steel surface. The advantage of the procedure is that it does not require prior knowledge about the groove geometry, and it is faster than the conventional RANSAC approaches for groove parametrization. The procedure is directly applicable to commercial laser triangulation sensors (where raw image data might not be available), which facilitates easier industrial implementation.

The rest of this paper is organized as follows. Section 2 presents the system under consideration, as well as the equipment and material used. Section 3 gives detailed derivations of the proposed method. Section 4 shows the experimental results, while the discussion on their significance and comparison of the proposed procedure and a more conventional RANSAC approach for line segment detection is given in Section 5. The conclusion and future work considerations are given in Section 6.

## 2. Materials and System Description

**Figure 1:**
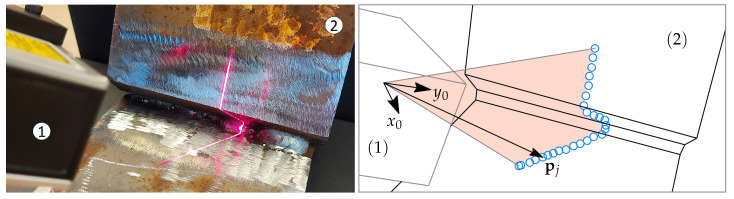
A commercial laser triangulation sensor (1) pointing at the weld groove of a test object (2). A local sensor fixed frame is denoted Frame 0 and all data points $p_{j}$ are obtained in the coordinates of Frame 0.

The system under consideration is a commercial line laser triangulation sensor and several different steel test objects with a weld groove between the plates, see Figure 1.

The sensor used in the tests is the Micro-epsilon scanCONTROL 2610-100 (produced by MICRO-EPSILON MESSTECHNIK). The sensor has a 658 nm red laser with 8 mW power output. The default settings are used without the pre-programmed cross-section templates. The output of the sensor is an array of 640 points in $R^{2}$, formatted as an array of 640 × 2 floating-point numbers.

**Figure 2:**
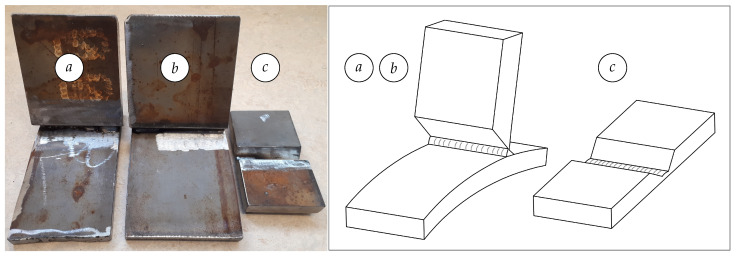
Three test objects used in the tests. The objects (*a*) and (*b*) represent a typical stub–joint groove, while the object (*c*) represents a typical butt-joint groove. The objects are welded with root welds of different widths. Schematic arrangement of the plates and the weld root pass in the test objects is shown to the right.

The test objects are steel plates (flat and cylindrical) welded together with different widths of the root welds and two different plate arrangements. The thickness of the flat plates is 22 mm and the thickness of the cylindrical plates is 20 mm, while the radius of the cylindrical plates is 600 mm. The material of the plates is S420G2+M according to EN 10225:2009. This is a typical material of tubular elements used for production of offshore jacket structures. The test objects are made to represent typical sections of tubular stub and butt joints. The first arrangement is typical for stub joints (*a*), (*b*) (where the leg plates are cylindrical) and the second arrangement is typical for butt joints (*c*) between large pipes, see Figure 2.

The test objects are ground similarly as they are in industrial situations, using the same type of power tools. The weld groove in the test objects (*a*) and (*b*) have different root conditions: from no root weld to step-wise increasing root weld. The test object (*c*) covers the cases with no root weld and a small (one weld pass) root weld. The scope of this work is limited to the cases with a ready-made root weld or with a backing weld, i.e., without a gap between plates. In practice, the root weld for very large pipes is expected to be welded manually due to large deviations in the pipe fit. Such root welds are preferred to be as small as possible (preferably one weld pass), but due to the larger gaps, they can require several weld passes, leading to wider root welds. Therefore, weld grooves with several different root weld widths are tested in this work.

In a typical industrial setup, such a sensor is fixed to a robot end-effector to perform scanning tasks. In this work, however, we propose the analysis algorithm for the obtained data, and, therefore, the sensor is hand-held for simplicity. The sensor obtains data points given in the coordinates of the sensor fixed frame, which is denoted Frame 0. This frame is oriented such that the xy-plane lies in the projected laser plane.

The goal of this work is to process the obtained data points and parameterize the weld groove. The parametrization is done in terms of the corner points of the weld groove.

## 3. Theoretical Basis of the Method

### 3.1. Geometrical Definitions

In this section, we present the geometrical objects which are used in the proposed algorithm. The main properties of those objects are also presented.

**Figure 3:**
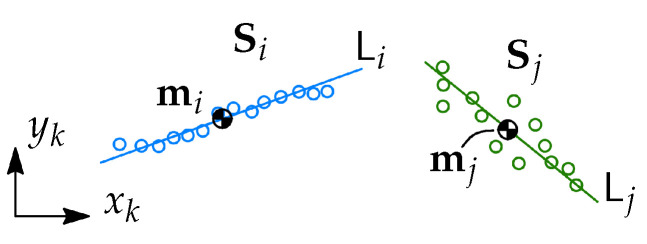
Two examples of point sets $S_{i}$ and $S_{j}$. Each of the point sets has the center of mass $m_{i}$ or $m_{j}$ and the line fit $L_{i}$ or $L_{j}$. The point sets are marked with different color and the line fit is shown using the same colors.

The first object we define in this section is a set of points (or a point set). The examples of point sets are given in Figure 3, where different colors are used to distinguish between them. A point set *i* given in the coordinates of Frame *k* is defined as

(1) $$\begin{array}{c} S_{i}^{k}=\left[\begin{array}{cccc} p_{i1}^{k} & p_{i2}^{k} & \cdots & p_{in_{i}}^{k} \end{array}\right]\in R^{2\times n_{i}} \end{array}$$

where $p_{ij}^{k}$ is the *j*-th point in the set *i* given in the coordinates of Frame *k* and $n_{i}$ is the number of points in the set.

The set (1) can be expressed in the coordinates of Frame *l* as

(2) $$\begin{array}{c} S_{i}^{l}=R_{k}^{l}S_{i}^{k} \end{array}$$

where $R_{k}^{l}\in \mathrm{SO}\left(2\right)$ is a rotation matrix from Frame *l* to Frame *k* [29]. The set $S_{i}^{k}$ has a center of mass point (CoM), which, when given in the coordinates of Frame *k*, is defined as

(3) $$\begin{array}{c} m_{i}^{k}=\frac{\sum_{j=1}^{n_{i}}p_{ij}^{k}}{n_{i}}. \end{array}$$

The point set $S_{i}^{k}$ is also equipped with a line fit obtained by linear regression, such a line fit is denoted $L_{i}^{k}$. The line fit can be represented as a typical line equation $y=a_{i}x+b_{i}$, where $a_{i}$ is the first-order coefficient, and $b_{i}$ is the zero-order coefficient.

**Figure 4:**
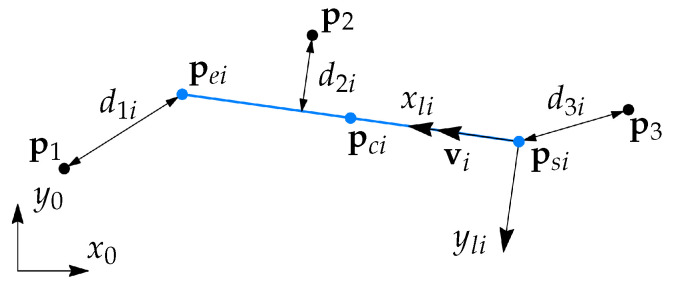
A line segment *i* is shown in blue color, with three different cases of point-to-segment distances $d_{ji}$ for j=1,2,3.

The main object used in the RANSAC search (presented in Section 3.2) is a 2-dimensional (2D) line segment. A line segment *i* (see Figure 4) is defined by the start point $p_{si}$ and the end point $p_{ei}$, while the center point is defined as $p_{ci}=0.5\left(p_{si}+p_{ei}\right)$.

A line segment has the unit direction vector $v_{i}$, the length $l_{i}$, and the local coordinate Frame li. Frame li is located such that the *x*-axis is along with $v_{i}$, and $p_{si}$ is the origin. An important property used in the RANSAC search is a point-to-segment distance used to identify inliers. The distance from the point $p_{j}$ to the segment *i* is defined as follows

(4) $$\begin{array}{c} d_{ji}=\left\{\begin{array}{cc} |p_{j,y}^{li}|, & \mathrm{if}\;p_{j,x}^{li}\geq 0\;\mathrm{and}\;p_{j,x}^{li}\leq l_{i}\\ ∥p_{j}^{li}∥, & \mathrm{if}\;p_{j,x}^{li}<0\\ ∥p_{j}^{li}-p_{ei}^{li}∥, & \mathrm{if}\;p_{j,x}^{li}>l_{i} \end{array}\right. \end{array}$$

where $p_{j}^{li}$ is a point in the coordinates of li, $p_{j}^{li}={\left[p_{j,x}^{li},\;p_{j,y}^{li}\right]}^{T}$, ∥·∥ is the Euclidean norm and |·| is the absolute value operator. In addition to the point-to-segment error, we will also use point-to-line errors attributed to segment *i*. This means that a line is defined using $p_{si}$ and $p_{ei}$, and the shortest distance from a point to that line is used as an error. A point-to-line error for a segment *i* is defined as

(5) $$\begin{array}{c} d_{l,ji}=\left|p_{j,y}^{li}\right|,\;\forall \;p_{j,x}^{li}. \end{array}$$

**Figure 5:**
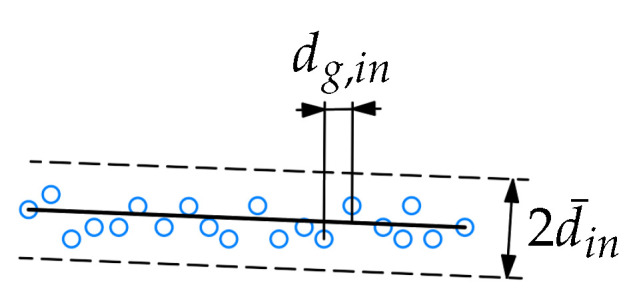
Inliers for a line segment with the inlier tolerance ${\bar{d}}_{in}$. The gap between points along the $x_{0}$ axis is denoted $d_{g,in}$.

The points located close to the line segment are classified as inliers if the distance to the segment $d_{in}$ is less or equal to the inlier tolerance ${\bar{d}}_{in}$, see Figure 5.

In this work, we use the point-to-line error metrics (5) in the initial RANSAC search (Section 3.2.1), and point-to-segment error metrics (4) in the iterative error elimination step (Section 3.2.4).

**Figure 6:**
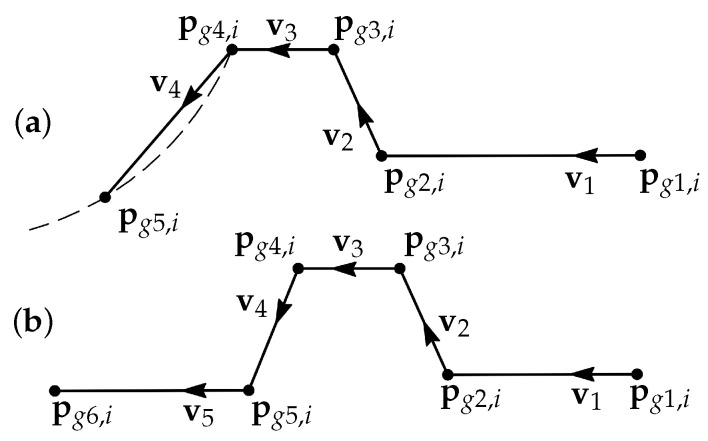
Two models for groove profiles: (**a**) a T-joint (i.e., stub joint) groove and (**b**) a butt joint groove (a V-groove) with a root weld. A stub groove and a butt groove consist of five or six corner points, respectively, which can also be represented as four or five sequentially connected line segments. The vectors $v_{i}$ show the directions of the segments.

Another object used in this work is a T-joint (or a stub joint) 2D groove profile and a V-groove profile from a butt joint. A typical model describing a groove profile with a root weld is shown in Figure 6.

A weld groove *i* consists of the corner points $p_{gj,i}$, which can also be represented by the sequentially connected line segments. For a stub joint groove it leads to the following definition of segment start and end points

(6) $$\begin{array}{cc} p_{s1} & =p_{g1,i},\\ p_{ej} & =p_{sj+1}=p_{gj+1,i}\;\;\mathrm{for}\;j=1,2,3,\\ p_{e4} & =p_{g5,i}. \end{array}$$

The segment points for a butt joint groove are defined similarly.

### 3.2. Detection of Weld Groove Corners from Noise-Free Data

The corner detection method presented in this section is a three-step algorithm. In the first step, the sequential RANSAC procedure for finding line segments is implemented. This gives a roughly good fit, which has varying quality due to the random nature of RANSAC. In the second step, after each completed RANSAC search, it is checked if a found segment has assigned corner points belonging to the next segment; if so, those points are removed, and the segment is updated. An assigned point is a point defined as an inlier of the current segment and is removed from the remaining points of the dataset. In the third step, an iterative error elimination algorithm is implemented for the entire groove profile model. This step provides the final correction of the RANSAC fit.

#### 3.2.1. Sequential RANSAC

The sequential RANSAC for detecting line segments from the sensor data is implemented utilizing the expected data structure. Specifically, we use the fact that all line segments should be sequentially connected and each point from the dataset has a unique $x_{0}$ coordinate. The line segments are searched in the direction from negative to positive $x_{0}$.

**Figure 7:**
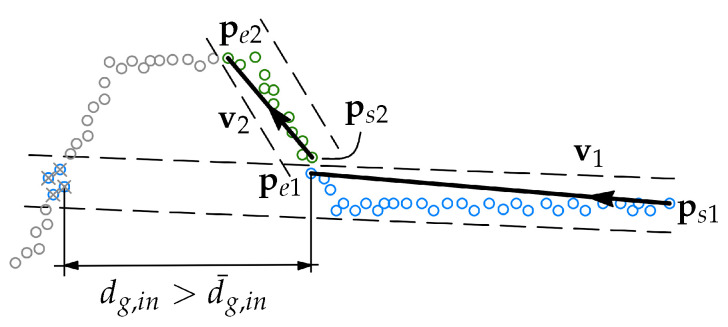
Procedure of searching the two first line segments using RANSAC. Inliers of segment 1 are shown in blue. These inliers, excluding the inliers separated by the gap $d_{g,in}>{\bar{d}}_{g,in}$, are used for defining the segment. After segment 1 is defined, the best inlier set for segment 2 is found, which is shown in green, and segment 2 is defined.

The procedure starts by assigning a start point for the first segment $p_{s1}$, which is the point with the smallest $x_{0}$ coordinate. Then a random point $p_{r}$, chosen through several iterations until the line fit with the most inliers, is found, see Figure 7.

The segment is then defined by the first and the last points in the inlier set, where the inliers after the first detected gap along the $x_{0}$ axis $d_{g,in}$ (see Figure 5) larger than the gap tolerance ${\bar{d}}_{g,in}$ are not included in the segment definition. Then, the next point after $p_{e1}$ is assigned to be $p_{s2}$, all inliers of segment 1 are removed from the dataset, and the RANSAC search is done for segment 2. This loop is repeated until the condition for the minimum number of remaining points in the dataset is met.

#### 3.2.2. Merging and Intersecting Segments

**Figure 8:**
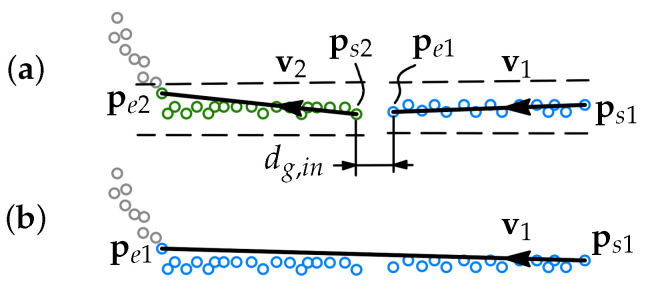
(**a**) Two segments 1 and 2 are found. The segments are divided since the gap $d_{g,in}>{\bar{d}}_{g,in}$. (**b**) If the angle between $v_{1}$ and $v_{2}$ is less than the defined angle tolerance ${\bar{\alpha }}_{12}$, then the two segments are merged.

The direction vector $v_{i}$ of every newly found segment *i* is compared to the direction vector $v_{i-1}$ of segment i−1. Segments *i* and i−1 are merged if the angle between the direction vectors $\alpha_{i,i-1}$ is small (i.e., within the defined tolerance ${\bar{\alpha }}_{i,i-1}$). This is demonstrated for segments 1 and 2 in Figure 8.

**Figure 9:**
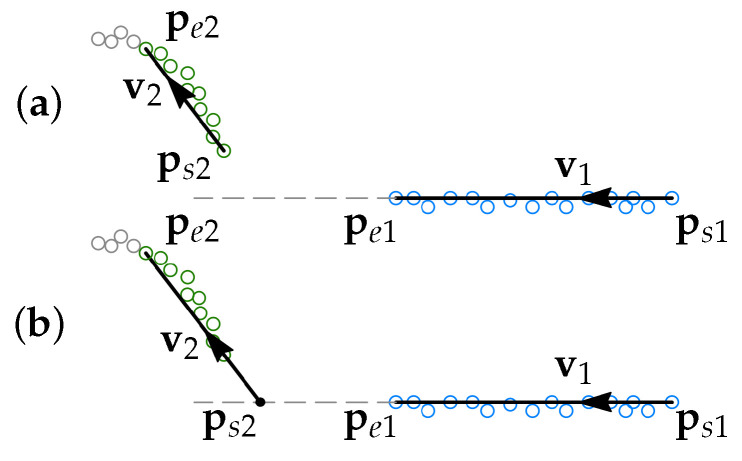
(**a**) Two segments 1 and 2 are found. (**b**) If the angle $\alpha_{12}\geq {\bar{\alpha }}_{12}$, then the start point $p_{s2}$ is moved to the intersection between the segments 1 and 2.

If the angle $\alpha_{i,i-1}$ is outside the tolerance ${\bar{\alpha }}_{i,i-1}$, then the point of intersection between segments *i* and i−1, $p_{int,i,i-1}$, is found and the start point of *i* is reset as $p_{si}=p_{int,i,i-1}$, see Figure 9.

#### 3.2.3. Correction of Assigned Corners

All points in the set of inliers attributed to a certain line segment can be referred to as points *assigned* to that line segment. The assigned points are removed from the overall dataset and will not be used in the further segment search.

**Figure 10:**
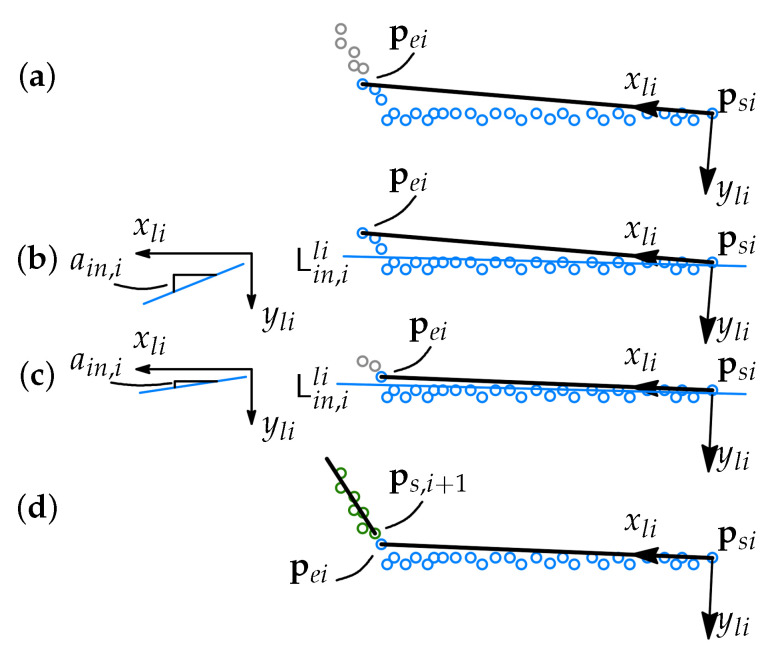
(**a**) Segment *i* and its local Frame li are defined over the inlier set, (**b**) A line fit $L_{in,i}^{li}$ for the inliers is found and the $a_{in,i}$ coefficient of the line equation is evaluated, (**c**) several points are removed from the inlier set and a line fit is evaluated again, (**d**) when $a_{in,i}$ is within the tolerance, segment *i* is defined over the rest of the inliers.

Consider a line segment *i* is being found. Then there are some points belonging to segment i+1 that will be assigned to segment *i* due to the inlier tolerance ${\bar{d}}_{in}$. Those points are referred to as assigned corner points or just an assigned corner. If segment i+1 is relatively short, then a corner assigned to segment *i* can cause problems searching segment i+1. This, for example, is a common situation for grooves with a narrow root weld. To avoid the above-described problem, we propose an intermediate step in the search algorithm, where the corner points assigned to segment *i* are returned to the overall dataset. The procedure is graphically demonstrated in Figure 10.

First, the line segment *i* is found (Figure 10a). Then the inlier coordinates are converted to the local coordinate system of the segment, the coordinates of the points are normalized along the *x* axis, and a line model fit is found (Figure 10b). If a corner of the next element is assigned, then the absolute value of the first-order coefficient of the line model is $|a_{in,i}|>0$. Then the inliers are removed from the end of the array until the acceptable limit of $a_{in,i}$ is reached (Figure 10c). In the final step, the line segment is reformulated using the newly defined last assigned point (Figure 10d), and the RANSAC search of the next line segment starts.

#### 3.2.4. Iterative Error Elimination

**Figure 11:**
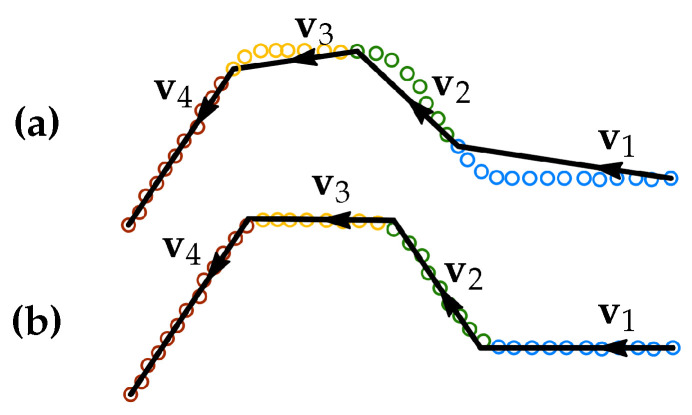
Input data (**a**) and the output (**b**) of the iterative error elimination algorithm. The different point colors indicate point sets associated with different segments, initial point association is shown in (**a**) and after the final iteration—in (**b**).

In the previous data analysis steps, line segments and groove corner points were defined sequentially by analysing only one segment at a time. It is, however, possible to improve the groove profile fit when the entire profile model is analyzed together with the dataset taking into consideration the relative geometric connections between segments. Therefore, we propose an iterative corner error elimination step for the entire groove model. The schematic representation of the input data with rounded corners and the groove profile fit before and after the iterative error elimination is shown in Figure 11.

**Figure 12:**
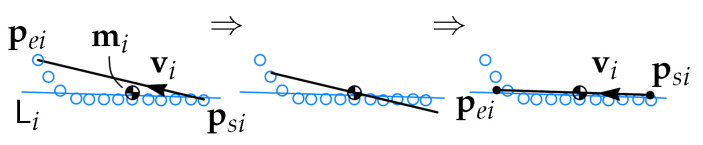
Alignment of segment *i* to the point set $S_{i}$. First the segment is translated to the mass center $m_{i}$ of $S_{i}$, then it is rotated to match the direction of $L_{i}$.

First, the points are divided into point sets depending on the segment that is the closest, i.e., the point *j* belongs to the point set *i* when

(7) $$\begin{array}{c} p_{j}\in S_{i}\;\mathrm{if}\;d_{ji}=\min\left(d_{ji}\right)\forall i \end{array}$$

where $d_{ji}$ is given in (4) and $\min(d_{ji})\forall i$ is the operator which returns minimum $d_{ji}$ for all *i*. For each point set *i* the CoM $m_{i}^{0}$ and the line fit $L_{i}^{0}$ are obtained, both in the coordinates of Frame 0, see Figure 12.

Then segment *i* moved by the vector

(8) $$\begin{array}{c} e_{i}^{0}=R_{li}^{0}\left[N_{y}R_{0}^{li}\left(m_{i}^{0}-p_{ci}^{0}\right)\right] \end{array}$$

where $p_{ci}^{0}$ is given in Figure 4 and

(9) $$\begin{array}{c} N_{y}=\left[\begin{array}{cc} 0 & 0\\ 0 & 1 \end{array}\right]. \end{array}$$

After the translation (8) the segment is rotated about $p_{ci}^{0}$ such that $v_{i}$ is aligned with $L_{i}$.

**Figure 13:**
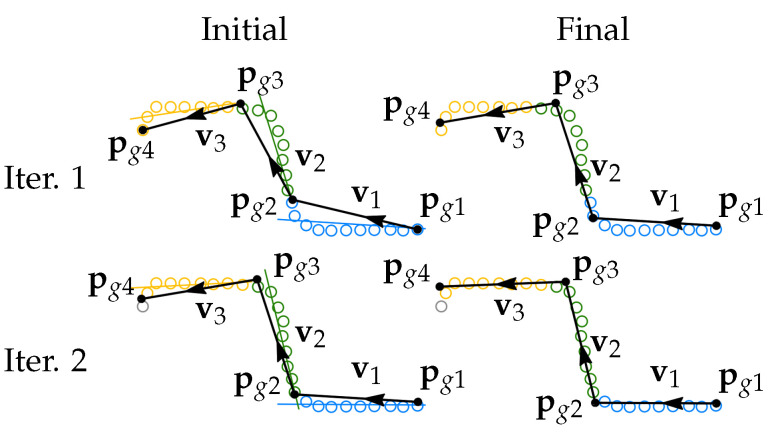
Schematic representation of the iterative corner error elimination algorithm. Two iterations are shown with the initial and final groove profile arrangements.

Consider a case with only three segments and two corners for simplicity, then two iterations of the above shown alignment are graphically shown in Figure 13.

The first iteration starts. The points are divided into point sets using (7), which is shown in three different colors. Then, the translation (8) (for i=1) and the rotation to the line fit $L_{1}$ is applied for segment 1. The start point of line segment 2 $p_{s2}$ is aligned with the new position of $p_{e1}$, and the translation (8) (for i=2) and the rotation to the line fit $L_{2}$ is applied for segment 2. The same operation is done for segment 3. The second iteration starts. The points are divided into point sets again, considering the new locations of the segments and the alignment is done for all three segments again. The procedure normally gives reasonable results after 4–5 iterations. Alternatively, a loop exit condition can be defined based on the convergence threshold for the position of the segment end points.

### 3.3. Detection of Weld Groove Corners from Noisy Data

This section further develops the procedure presented in Section 3.2. In this section, the data noise due to reflections in the polished steel surfaces is taken into account.

After each line segment is found, the algorithm performs a check if there is reflection noise after the line segment. If no noise is detected, the RANSAC search of the next line segment starts. If the noise is detected, then the first non-noisy point is sought, and the RANSAC search of the next line segment starts from that point. The details of the procedure are given in the following subsections.

#### 3.3.1. Noise Detection

In Section 3.2.1, it was described that the end of a line segment is determined by the gap between inliers being larger than the defined gap tolerance. This can happen due to partly missing data or when the data point deviates outside the inlier tolerance. This deviation can be just due to the shape of the point cloud (see Figure 8), and it can be caused by noise in the data. Therefore, after each line segment is found, a check for noise is performed.

**Figure 14:**
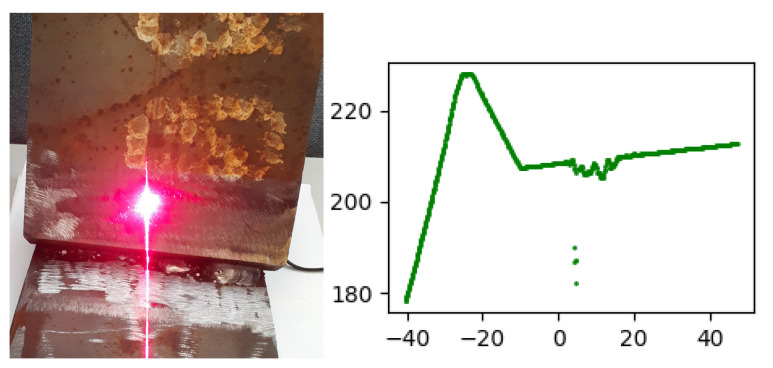
Reflections from laser projection onto shiny metal surface and the corresponding data noise in the graph.

When a laser is pointed at shiny reflective surfaces, the projected laser line is reflected (sometimes several times), producing light pollution around the true laser line projection, see Figure 14.

This light pollution causes difficulties for the sensor algorithm to identify the true laser line projection, and, therefore, such line pollution is referred to as noise in this work. The noise causes the data points to deviate from their true locations in the final 2D data output of the sensor. Such noise is rather difficult to predict, since it varies depending on the scanned surface finish and the geometry of the groove. However, arrays of consequent noisy points in most of the cases have different geometrical structures, comparing to the noise-free points. In this subsection we propose several metrics $M:R^{2\times \cdots }\to R$ which can be used to identify noisy points from the noise-free points.

The first noise detection check is based on the maximum relative $y_{0}$-distance between the two neighboring points. Consider that any point of $S_{i}^{0}$ has the coordinates $p_{ij}^{0}={\left[x_{ij}\;y_{ij}\right]}^{T}$, then

(10) $$\begin{array}{c} \Delta p_{ij}^{0}=\frac{y_{ij}-y_{i,j-1}}{x_{ij}-x_{i,j-1}} \end{array}$$

is the relative *y*-gap between the points, and the condition is given as

(11) $$\begin{array}{c} \max(∥\Delta p_{ij}^{0}∥)\;\;\forall j\backslash 1>{\bar{d}}_{n1} \end{array}$$

where ${\bar{d}}_{n1}$ is the relative gap condition tolerance. Another condition on the relative *y*-gap size inconsistency is defined as

(12) $$\begin{array}{c} \frac{\sum_{j=2}^{n_{i}-1}∥\Delta p_{ij}^{0}-\Delta p_{i,j-1}^{0}∥}{n_{i}-2}>{\bar{d}}_{n2}. \end{array}$$

**Figure 15:**
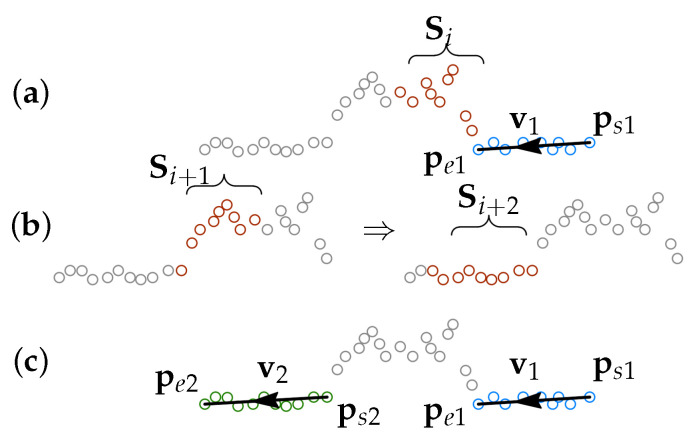
Schematic representation of the noise detection procedure. When segment 1 is found, the algorithm detects noise in the set of the following points $S_{j}$ (**a**), then the following point sets $S_{j+1}$ and $S_{j+2}$ are checked for noise (**b**), segment 2 is defined once a noise free set is found (**c**).

The noise detection checks are performed over a set of consequent points $S_{i}=\left[p_{i1}\;\cdots \;p_{in_{i}}\right]$, where the number of points in the set is a parameter, see Figure 15a.

**Figure 16:**
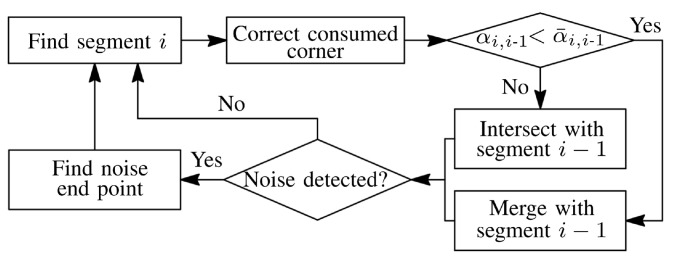
Flow diagram the proposed procedure for groove parametrization with noisy data points. The diagram does not include the iterative error elimination step.

If the noise is detected in the set $S_{i}$, then a new set $S_{i+1}$ is checked for noise, see Figure 15b. The first point in the set $S_{i+1}$ is selected to be $p_{i+1,1}=p_{in_{i}}$. This process continues until a noise-free set is found. When the first noise-free point set is found, then the first point of the set is used for the RANSAC search of the next line segment, see Figure 15c. The flow diagram of the procedure is shown in Figure 16.

#### 3.3.2. Iterative Error Elimination for Noisy Data

The iterative error elimination procedure for noisy data is essentially the same as described in Section 3.2.4. However, the difference is that all the points from the noisy point sets $S_{i}^{0}$ are removed from the original dataset. Then the iterative error elimination procedure is initiated.

It is worth noting that if the data contains a lot of noise, then the iterative error elimination procedure can result in divergence from the best-fit groove corner position. This happens due to a lack of data points, which gives a poor line fit. This means that the procedure should be avoided when very noisy data are analyzed.

## 4. Experimental Results

In this section, the performance and precision of the weld groove parametrization algorithm are studied using the data obtained experimentally by scanning the test objects shown in Figure 2. The performance of the proposed algorithm is evaluated using two parameters. The first one is the execution time $t_{ex}$ (wall-clock time is used). The second one is the average relative distance $d_{e,i}$ of the points in $S_{i}$ to the found segment *i*, where $S_{i}$ is a set of points with the shortest distance (4) to segment *i* compared to the other segments (7). Both the execution time and the fit error were calculated for 10 consequent runs of the algorithm to simulate scanning multiple sections along the weld groove. The algorithm is implemented in Python and is run on a notebook computer with the Ubuntu operating system, 2-core Intel Core i7 CPU, and 16 GB of RAM.

The data were sampled by a hand-held sensor. The orientation of the sensor was selected and altered such that it was possible to capture various types of noise, as well as collect the data without noise. First, the scanned data without noise is analyzed and the discussion on different functionality of the proposed groove parametrization procedure is presented. Then, the data with various typical types of noise caused by reflections are analyzed. Three main noise groups are defined in this work: N1—noise on the outer surfaces, N2—noise in the corners, and N3—noise inside the groove. The datasets analyzed are summed up in Table 1.

### 4.1. Results Using Noise-Free Data

#### 4.1.1. Stub Joints

The scope of this part of the experimental program was to demonstrate the performance and speed of execution of the proposed groove parametrization algorithm when applied to stub joint grooves. The root widths of 0, 3, and 11 mm were considered.

**Figure 17:**
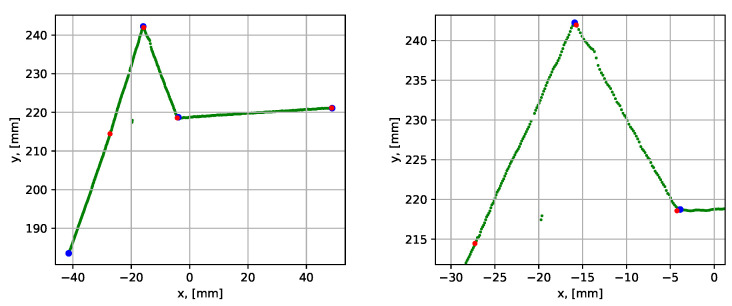
Parametrization results with groove corner points for dataset 1. The blue dots show the result before the iterative error elimination procedure and the red dots show after. A close-up of the graph is shown to the right.

First, the performance of the groove parametrization algorithm was studied using the case of a groove without a root weld (i.e., 0 mm root weld), which was a relatively simple case. The scanned data were denoted as dataset 1. The dataset was of good quality with distinct sharp corners, which gave good groove parametrization results, see Figure 17.

The blue corner dots in the graph show the result before the iterative error elimination procedure (see Section 3.2.4) and the red dots show after. The last segment of the section was shortened to 30 mm, and then the iterative error elimination step was applied. This was necessary since the last side of the groove was curved (due to the geometry of stub joints), and the best line fit was obtained locally in the groove. The quantitative evaluation of the parametrization results is given in Table 2, which shows maximum, minimum, and mean execution times and segment fit errors for 10 consequent algorithm runs for the same dataset.

The mean execution time was measured to be 0.061 s, which gives above 16 scans per second. Provided that the entire weld needs to be scanned discretely every 20–30 mm along its length, such execution speed would allow for an approximate scanning capacity of 400 mm/s. Such scanning speed is several times above the practical scanning speed in the industry. The average point-to-segment error among all segments was within 0.034 to 0.070 mm, which was a very good segment-to-data fit. It is also notable that the maximum, minimum, and mean results were consistent over 10 algorithm runs due to the good quality of the scanned data.

**Figure 18:**
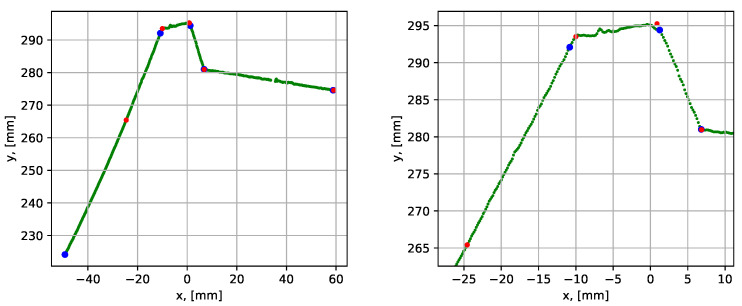
Parametrization results with groove corner points for dataset 2. The blue dots show the result before the iterative error elimination procedure and the red dots show after. A close-up of the graph is shown to the right.

The next analyzed dataset (dataset 2) was obtained by scanning the groove with a 10 mm root, see Figure 18.

The results showed a clear improvement of the groove parametrization after the iterative error elimination step, which is also reflected in Table 2. The mean execution time was 0.092 s, which was slightly higher than in the previous case. This was due to one extra segment compared to the case without a root weld. The average point-to-segment error after the iterative error elimination step was relatively consistent over 10 algorithm runs. However, the average point-to-segment error before the error elimination procedure was significantly higher and was inconsistent from run to run. This demonstrates the benefit of using the iterative error elimination procedure.

**Figure 19:**
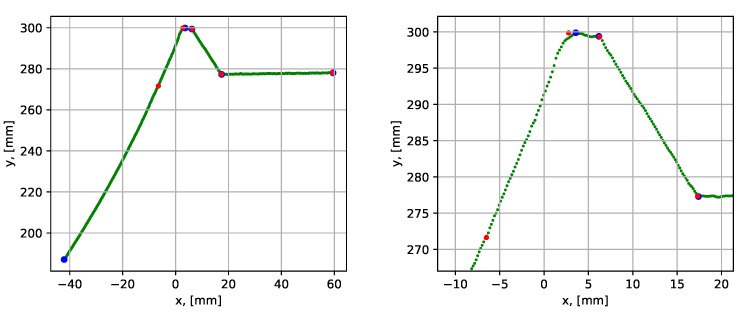
Parametrization results with groove corner points for dataset 3. The blue dots show the result before the iterative error elimination procedure and the red dots show after. A close-up of the graph is shown to the right.

The next test was performed for the groove with a 3 mm wide root weld (the dataset 3). Parametrization of grooves with a narrow root weld is a more complex task, since the algorithm can neglect the root by assigning its point as inliers to the neighboring groove sides. Therefore, the correction of assigned corners procedure (see Section 3.2.3) was introduced in the algorithm. This correction prevents the points of the next segment from being assigned as inliers of the current segment. The results of the parametrization are shown in Figure 19 and the quantitative results are given in Table 2.

**Figure 20:**
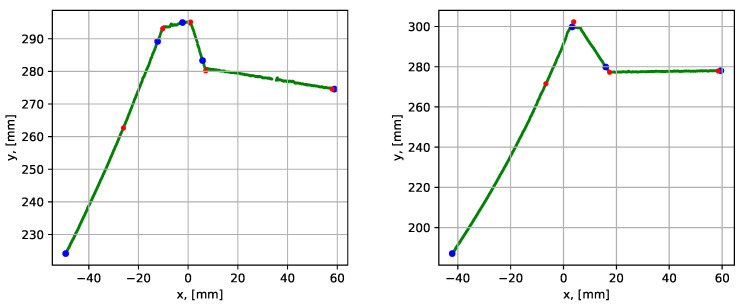
Parametrization results with groove corner points for dataset 2 (to the left) and 3 (to the right), when the correction of assigned corners step is omitted. The blue dots show the result before the iterative error elimination procedure and the red dots show after.

The mean execution time was 0.126 s, which roughly corresponded to 200 mm/s available scanning speed. The point-to-segment errors were similarly low compared to the previous tests. To demonstrate the importance of the correction of assigned corners procedure, the results for datasets 2 and 3 without the correction step are presented in Figure 20.

For the case with a 10 mm root, it is seen that the blue dots are located well after the corners. In that case, the iterative error elimination step corrected that error, moving the corner points to their realistic positions (the red dots). In the case with a 3 mm root (to the right), it is seen that the root segment was not detected at all, and the iterative error elimination procedure is not designed to correct such errors.

#### 4.1.2. Butt Joints

The weld groove parametrization procedure presented in this work was originally developed for stub joints. In fact, the procedure is independent of the groove configuration. In this subsection we demonstrate that it can also be efficiently applied for the weld grooves in butt joints.

**Figure 21:**
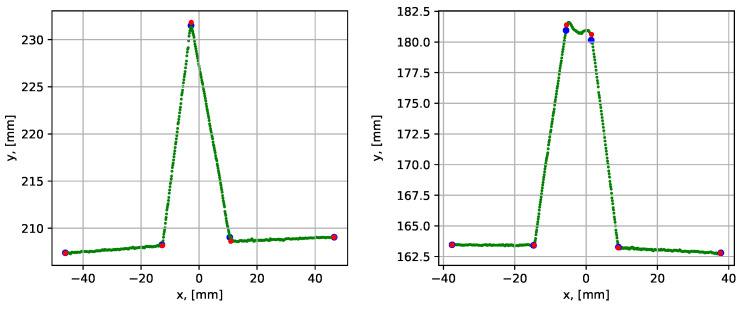
Parametrization results with groove corner points for datasets 4 (to the left) and 5 (to the right). The blue dots show the result before the iterative error elimination procedure and the red dots show after.

The tests with a butt joint groove were performed for the case without root weld (i.e., 0 mm root) and the case with 7 mm root weld, using datasets 4 and 5 (see Table 1). The results are given in Figure 21.

The execution times and average point-to-segment errors for 10 sequential runs are given in Table 3. The execution times were in accordance with the results obtained from scanning stub joint grooves, taking the number of segment into consideration. The average point-to-segment error was of a similar magnitude comparing to previous results, as well as the error was relatively consistent over the 10 algorithm runs. The slightly higher average error of 0.160 mm was observed for segment 3 (dataset 5) in Table 3 due to the convex shape of the root weld. The slightly higher inconsistency between the maximum and minimum values is also attributed to the same reason.

### 4.2. Results Using Noisy Data

In this subsection, we study the performance of the algorithm using datasets with noise caused by reflections. The data noise caused by reflections can be different in structure and intensity, and can be located in different places. This depends on the surface treatment and laser pointing angle. In this work, we used test objects with surfaces machined using the same type of power tools as used in the industry, and the laser pointing angle was selected freely to obtain datasets with various types of noise. Data noise was classified into 3 different groups (N1, N2, and N3) depending on the noise location. The groups are described in the introduction to Section 4. Two datasets from each noise group were tested in this part of the experimental program. The quantitative evaluation of the algorithm performance was given in terms of the maximum and mean values. Only root weld widths of 0 and 3 mm were considered, as the primary focus of this subsection is studying the performance of the algorithm with different types of noise. Only tests with stub joint grooves were considered here. The test results with butt joint grooves along with the comparison to a more conventional RANSAC groove parametrization algorithm will be presented in the next section.

**Figure 22:**
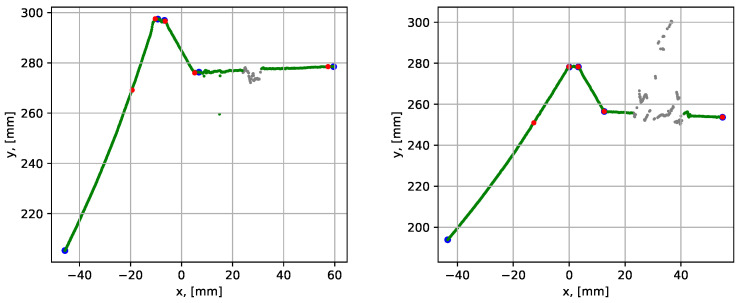
Parametrization results with groove corner points for datasets 6 (to the left) and 7 (to the right). The blue dots show the result before the iterative error elimination procedure and the red dots show after. The grey points have been identified as noise by the noise detection algorithm.

First, datasets 6 and 7 were analyzed, see Table 1. The data contained noise on the outer plate surfaces, i.e., the noise of type N1. The parametrization results are shown graphically in Figure 22, and the quantitative results are given in Table 4.

The grey color in the graphs indicates the points identified as noise by the noise detection algorithm. The mean execution time was within 0.126 to 0.139 s, which was similar to the results using the noise-free dataset 3. The average point-to-segment error among all segments was within 0.039 to 0.258 mm, which was higher than the errors using the noise-free datasets. This could be explained by the minor noise, which was within the segment inlier tolerances and was not identified as noise by the noise detection algorithm. Such minor noise caused larger distances from the points to the found segment, which led to larger point-to-segment errors. It is also notable that the maximum and mean results were relatively consistent over 10 algorithm runs.

**Figure 23:**
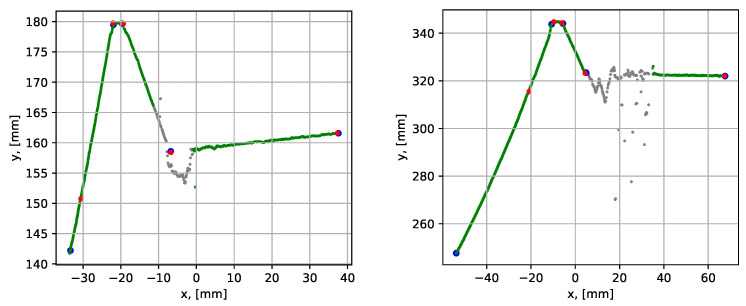
Parametrization results with groove corner points for datasets 8 (to the left) and 9 (to the right). The blue dots show the result before the iterative error elimination procedure and the red dots show after. The grey points have been identified as noise by the noise detection algorithm.

The next datasets analyzed (datasets 8 and 9) were selected based upon their containing corner noise (i.e., type N2). The results showed that it was possible to identify the corner noise and locate the missing groove corner, see Figure 23 and Table 4. The noise did not affect the mean execution times, which were within 0.061 to 0.108 s. The average point-to-segment errors were within 0.044 to 0.160 mm, which were of the same magnitude as in the previous cases.

**Figure 24:**
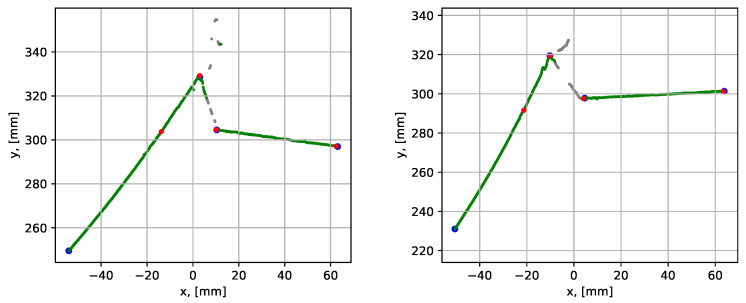
Parametrization results with groove corner points for datasets 10 (to the left) and 11 (to the right). The blue dots show the result before the iterative error elimination procedure and the red dots show after. The grey points have been identified as noise by the noise detection algorithm.

The two last noisy datasets (datasets 10 and 11) were selected to include the N3 noise, which is the noise inside the groove. The results are plotted in Figure 24. The weld groove, in this case, was without a root, which was selected based on groove grinding conditions in the available test objects giving the best examples of the N3 noise.

The N3 noise is different from the N1 and N2 types since it is mostly caused by the dominant part of spread reflections towards the other wall of the groove. This means that the noisy points are not necessarily located chaotically in the $y_{0}$ direction (see, for example, Figure 22). The noisy points can, alternatively, form rather well-structured line segments in the false locations, see the graph to the right in Figure 24. In this case, the only effective noise detection condition is (11). Regardless of the more complex noise detection problem, the execution times and the average point-to-segment errors were similar to all other cases considered in this subsection, see Table 4.

## 5. Discussion and Comparison to the Conventional RANSAC Algorithm

In this section a discussion on the advantages and disadvantages of the proposed procedure compared to the recently published methods is given. In addition, the proposed procedure is compared to a groove parametrization procedure based on the conventional RANSAC approach for line segment search.

Recently, several methods have been proposed for weld seam tracking, where weld seam or weld groove parametrization (feature extraction) was a part of the procedure. In most of the proposed methods groove parametrization was done at the camera image level, i.e., using camera image points or pixels [14,16,26,27]. Image points can be seen as raw data, which gives more flexibility in choosing filtering, processing and parametrization techniques. In our work the proposed procedure used triangulated sensor points for analysis, i.e., 2D points given in the coordinates of local sensor coordinate frame. This can be advantageous if access to raw image data is not possible, when commercial line laser sensors are used. Some methods using triangulated points have been also previously reported (e.g., [13]), however the methods did not include strong data noise caused by the reflections of a laser.

Processing noisy data has been an important part of the recent developments. It is, however, worth noting that the recent methods have mostly been made for online seam tracking where arc and splashing noise was a central problem. It is beneficial for robotic welding when scanning and welding can be done simultaneously. In the case of a stub joint, it is more reasonable to scan and parameterize the entire weld groove and then generate motion paths for a welding robot. This is justified by the fact that the interface between elements in a stub joint is rather complex, which leads to the groove with variable number of weld layers and beads along the weld. In such cases, it is more reasonable to plan the entire multi-pass weld before the welding of the first bead starts. At the same time a groove has to be ground just before welding to remove rust and dust. Then the noise caused by laser reflections is a dominant noise problem, while results in Section 4 revealed that the noise can be quite severe.

As in our work, a set of groove corner points is often selected as a parametrization basis in the previously reported methods. The dominant approaches for groove parametrization are the methods based on gradients, which can be both of the first-order or the second-order, or various combination of both [13,14,15]. These approaches proved to be fast enough to be used in real-time weld seam tracking. The results reported in Section 4 suggest that the proposed method is running at approximately 10 Hz, which can be too slow for some real-time seam tracking. On the other hand, due to the nature of data gradient functions, the obtained parametrization results might be poor for triangulated points with a lot of data noise.

The parametrization methods which are the closest to the proposed procedure are the more conventional RANSAC approaches for line segment detection [10,17]. RANSAC uses model fitting, and can be effective even with very noisy data. Therefore, RANSAC was selected as a basis for the proposed method. When used with noise-free data, RANSAC can effectively detect lines and line segments without prior knowledge of the weld groove geometry [17]. In the presence of strong data noise, RANSAC would also detect line segments from noisy points or line segments deviated by noise. Then, pre-knowledge of the groove geometry is required to filter out the line segments from noisy points. In this work we propose a procedure for the precise parametrization of weld grooves from triangulated points with strong data noise, and, therefore, we suggest that comparing our approach with the conventional RANSAC line segment detector is a relevant addition to our experimental investigation. The comparison is presented in the rest if this section.

Summing up, the advantages of the proposed procedure are:

- Can be used with commercial sensors where only triangulated data points are available;
- Can be used with strong data noise, cause by laser reflections;
- Can be used for grooves in both stub and butt joints;
- Does not require pre-knowledge of the weld groove geometry,

while the disadvantages compared to the recent works are:

- Might be too slow for some real-time robot path generators;
- Does not account for a gap between plates;
- Is not developed to filter out arc and splash noise.

Given the advantages and disadvantages above, the proposed procedure is relevant in terms of the industrial implementation as a part of the robotic welding system for large tubular joints.

### 5.1. Conventional RANSAC Algorithm

This method is based on the conventional RANSAC approach for searching line segments. Therefore, a term *conventional* was adopted. The algorithm used here is similar to that implemented in [17], with some more logic implemented for analyzing grooves with narrow root welds and filtering out the line segment found from noisy points. The procedure consists of the following steps. First, a point pair is randomly selected from the entire dataset, then a line is defined through the selected points. Next, the points within the defined margin to the line (inliers) are identified, and the largest line segment on the line is found. These steps are repeated for a number of iterations until the segment with the most inliers is found, then a line segment is defined using linear regression for the best inlier set. Once all line segments are found (i.e., the condition for min number of the remaining points is met), groove corners are found as intersections between the line segments, which correspond to the pre-defined groove opening angle. Additional logic is implemented to search for a root weld in a groove before the groove side walls, This way, it is ensured that narrow root welds will not be neglected. It is noted that the conventional RANSAC algorithm does not need pre-knowledge of groove geometry to detect line segments. That knowledge is, however, required for filtering out the line segments detected from noisy points.

### 5.2. Comparison of Experimental Results

Comparison of experimental results from the proposed and conventional RANSAC procedures was made using an example of a V-groove, which is typical for butt joints, which means that the test object c) was used (see Figure 2). The results are compared in terms of the execution time and average point-to-segment errors.

Comparison of the results for dataset 5 was made using the mean dataset 5 values only from Table 3 and the mean results using the conventional RANSAC approach, which is summed up in Table 5.

It is noted that for the other datasets used for the comparison, we will not provide the explicit results with the maximum, minimum, and mean values, but rather give a similar summary table with mean values only. The results given in Table 5 showed a clear advantage of the proposed method in terms of the execution time, which was 0.142 s compared to 1.886 s from the conventional approach. The execution time was the average value per run over 10 consequent runs. The precision of the parametrization in terms of the average point-to-segment error was rather similar. The proposed method gave a better precision for segments 2 to 5, while the conventional method gave a better precision for segment 1. It is, however, noted that both methods had high precision in the results, and minor numerical variations did not have practical importance for the results. It was concluded that the proposed method had the same precision as the conventional method for the tested noise-free dataset, while the execution time was more than 10 times faster.

**Figure 25:**
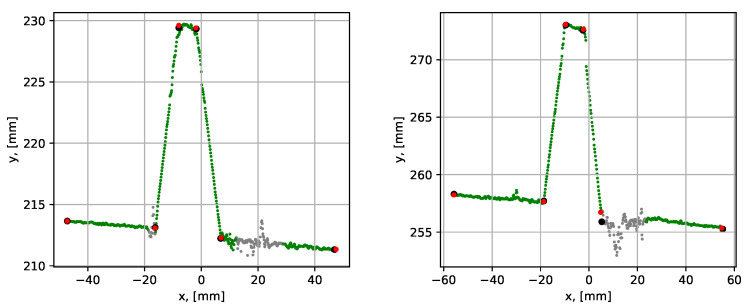
Parametrization results with groove corner points for datasets 12 (to the left) and 13 (to the right). The red dots show the result from the proposed procedure and the black dots show results from the conventional RANSAC. The grey points have been identified as noise by the noise detection algorithm.

Next, the performance of the proposed and conventional RANSAC algorithms was compared using the noisy datasets 12 and 13 (see Table 1), which covered noise cases N1 and N2 (outer surface and corner noise). The graphical comparison of the results is given in Figure 25 and the numerical comparison of the results is given in Table 6.

The mean execution time over 10 consequent runs for the proposed method was within 0.082 to 0.095 s, while for the conventional RANSAC method, the execution time was within 1.258 to 1.858 s. The presence of data noise led to a larger variation in average point-to-segment errors. It is, however, again highlighted that both methods could be regarded as similarly precise since the errors were small in both cases.

**Figure 26:**
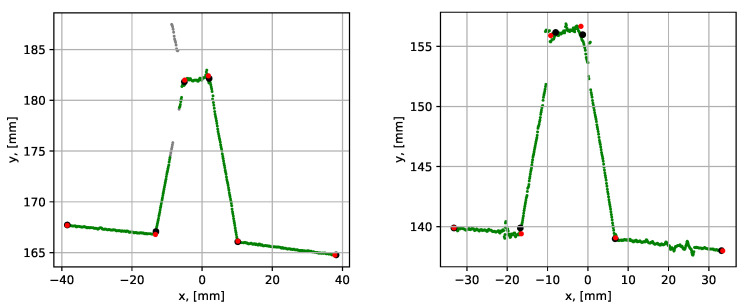
Parametrization results with groove corner points for datasets 14 (to the left) and 15 (to the right). The red dots show the result from the proposed procedure and the black dots show results from the conventional RANSAC. The grey points have been identified as noise by the noise detection algorithm.

The last comparison study was performed using datasets 14 and 15, where typical N3 noise was present. The results of the comparison are given in Figure 26 and Table 6.

A similar difference, as for the previous cases, in the execution times was also observed for the current study. The mean execution time for the proposed method was 0.087 s compared to 1.205–1.665 s from the conventional RANSAC method. The average point-to-segment error was of the same magnitude as in the previously studied cases. It is noted that the average errors below 0.5 mm can be considered as a good fit, which is well satisfied by both methods seeing from the results in Table 6.

The comparison study can be summed up with the following conclusions. Both the proposed and conventional RANSAC approaches gave similarly high precision for the noise-free and noisy datasets. The average execution time was, however, more than 10 times faster for the proposed method. The difference can be explained by the fact that the proposed method uses less iterations to find the segments, since the first point of the segment is defined. In addition, the conventional RANSAC approach uses many iterations for searching the root segment first. The root segment has relatively few points, and the algorithm does not proceed to search the other segments before the root is found, which leads to longer execution times. It is also noted that the conventional RANSAC approach needs the approximate groove geometry as an input, while the proposed method can do parametrization without any knowledge of the groove geometry.

## 6. Conclusions and Future Work

We have presented a novel weld groove parametrization procedure for typical grooves in large tubular butt and stub joints. The procedure was specifically developed to be used with industrial 2D laser scanners where raw image data might not be available, facilitating easier industrial implementation. The procedure consisted of a combination of a sequential RANSAC search and the two additional correction steps for increasing the precision of parametrization. In addition, the data noise detection steps were introduced to enable processing of noisy data, where the noise is caused by reflections between the groove surfaces. The performance of the procedure was studied experimentally using three test objects with grooves machined similarly as in the industrial applications. Two objects represented a stub joint groove, and one object represented a butt joint groove. The procedure proved to be efficient for both types of weld grooves using noise-free data. The mean execution time was within 0.061 to 0.142 s, corresponding to 170 to 400 mm/s available scanning capacity (25 mm between scans). In the cases with data noise, the parametrization results for both grooves were still of sufficient quality, while the mean execution time was within 0.046 to 0.139 s, which was similar to the noise-free cases. The performance of the proposed procedure was also compared to the conventional RANSAC approach for line segment search. The proposed method was as precise as the conventional RANSAC. However, the mean execution time was more than 10 times faster for the proposed method. Additionally, the proposed method did not require any prior knowledge of the groove geometry. The contribution of this work is a step towards robotic scanning and weld path planning for large tubular joints. Therefore, a natural topic for future work is studying the performance of the proposed procedure using a robot-fixed sensor and a full-scale tubular joint. In addition, scanning strategies facilitating the reduction in reflections and data noise should be investigated.

## Figures and Tables

**Table 1:** Datasets from the scanned weld grooves.

No.	Type	Root,	Noise	No.	Type	Root,	Noise	No.	Type	Root,	Noise
		[mm]				[mm]				[mm]	
1	Stub	0	No	6	Stub	3	Low N1	11	Stub	0	Medium N3
2	Stub	11	No	7	Stub	3	High N1	12	Butt	7	Medium N1
3	Stub	3	No	8	Stub	3	Medium N2	13	Butt	7	Medium N2
4	Butt	0	No	9	Stub	3	High N2	14	Butt	7	Medium N3
5	Butt	7	No	10	Stub	0	Low N3	15	Butt	7	Low N3

**Table 2:** Segment fit errors and execution times for 10 sequential runs using the datasets 1, 2, and 3. Results for dataset 2 are given with and without iterative error elimination step (IEES). The fit error definition is given in the beginning of Section 4.

Dataset	1			2 with IEES			2 without IEES			3		
	Max	Min	Mean	Max	Min	Mean	Max	Min	Mean	Max	Min	Mean
Time, [s]	0.076	0.043	0.061	0.125	0.056	0.092	-	-	-	0.145	0.058	0.126
$d_{e,1}$, [mm]	0.034	0.034	0.034	0.064	0.061	0.062	0.062	0.062	0.062	0.056	0.056	0.056
$d_{e,2}$, [mm]	0.070	0.070	0.070	0.114	0.095	0.109	1.058	0.040	0.192	0.041	0.041	0.041
$d_{e,3}$, [mm]	0.050	0.050	0.050	0.123	0.115	0.118	1.152	0.122	0.740	0.095	0.095	0.095
$d_{e,4}$, [mm]	-	-	-	0.046	0.044	0.044	0.659	0.400	0.618	0.074	0.074	0.074

**Table 3:** Segment fit errors and execution times for 10 sequential runs using datasets 4 and 5. The fit error definition is given in the beginning of Section 4.

Dataset		4			5	
	Max	Min	Mean	Max	Min	Mean
Time, [s]	0.138	0.062	0.091	0.233	0.093	0.142
$d_{e,1}$, [mm]	0.030	0.028	0.030	0.062	0.030	0.046
$d_{e,2}$, [mm]	0.074	0.074	0.074	0.077	0.026	0.052
$d_{e,3}$, [mm]	0.036	0.035	0.036	0.161	0.160	0.160
$d_{e,4}$, [mm]	0.032	0.032	0.032	0.042	0.041	0.041
$d_{e,5}$, [mm]	-	-	-	0.019	0.019	0.019

**Table 4:** Segment fit errors and running times for 10 sequential runs using the datasets 6–11. The fit error definition is given in the beginning of Section 4.

Dataset	6		7		8		9		10		11	
	Max	Mean	Max	Mean	Max	Mean	Max	Mean	Max	Mean	Max	Mean
Time, [s]	0.133	0.126	0.262	0.139	0.089	0.061	0.133	0.108	0.162	0.125	0.066	0.046
$d_{e,1}$, [mm]	0.258	0.258	0.178	0.115	0.086	0.086	0.106	0.105	0.077	0.077	0.070	0.070
$d_{e,2}$, [mm]	0.144	0.141	0.040	0.040	0.078	0.076	0.050	0.047	0.122	0.120	0.086	0.086
$d_{e,3}$, [mm]	0.229	0.220	0.108	0.11	0.062	0.044	0.160	0.160	0.102	0.102	0.120	0.120
$d_{e,4}$, [mm]	0.077	0.077	0.039	0.039	0.049	0.048	0.157	0.157	-	-	-	-

**Table 5:** Comparison of the results using dataset 5. The fit error definition is given in the beginning of Section 4.

Mean Values	Proposed	Conventional
Time, [s]	0.142	1.886
$d_{e,1}$, [mm]	0.046	0.037
$d_{e,2}$, [mm]	0.052	0.062
$d_{e,3}$, [mm]	0.160	0.164
$d_{e,4}$, [mm]	0.041	0.042
$d_{e,5}$, [mm]	0.019	0.036

**Table 6:** Comparison of the results using datasets 12–15. The fit error definition is given in the beginning of Section 4.

Dataset	12		13		14		15	
Mean Values	Prop.	Conv.	Prop.	Conv.	Prop.	Conv.	Prop.	Conv.
Time, [s]	0.095	1.858	0.082	1.258	0.087	1.205	0.087	1.665
$d_{e,1}$, [mm]	0.106	0.155	0.077	0.068	0.025	0.080	0.082	0.146
$d_{e,2}$, [mm]	0.105	0.092	0.133	0.057	0.060	0.095	0.154	0.256
$d_{e,3}$, [mm]	0.151	0.241	0.061	0.072	0.152	0.153	0.135	0.277
$d_{e,4}$, [mm]	0.078	0.100	0.030	0.117	0.044	0.078	0.303	0.151
$d_{e,5}$, [mm]	0.035	0.115	0.069	0.394	0.021	0.060	0.090	0.103
